# Acknowledgment to the Reviewers of *Journal of Fungi* in 2022

**DOI:** 10.3390/jof9020137

**Published:** 2023-01-19

**Authors:** Journal of Fungi Editorial Office

**Affiliations:** MDPI AG, St. Alban-Anlage 66, 4052 Basel, Switzerland

High-quality academic publishing is built on rigorous peer review. *Journal of Fungi* was able to uphold its high standards for published papers due to the outstanding efforts of our reviewers. Thanks to the efforts of our reviewers in 2022, the median time to first decision was 15 days and the median time to publication was 34 days. Regardless of whether the articles they examined were ultimately published, the editors would like to express their appreciation and thank the following reviewers for the time and dedication that they have shown *Journal of Fungi*:
Abdel Latef, Arafat Abdel HamedLiu, GangAbdel-Azeem, Ahmed MohamedLiu, TingAbdelmohsen, Usama RamadanLiu, TongbaoAbdel-Raheem, M. A.Liu, ZhengchangAbd-Elsalam, Kamel AhmedLiu, ZhonghuaAbe, MasahiroLiu, ZhongweiAbghari, AliLizarazo, JairoAbiri, RambodLlama-Palacios, AranchaAbo-Elyousr, Kamal A. M.Lo, Hsiu-JungAbou Fayssal, SamiLoc, Nguyen HoangAbrantes, Ruben A.Lockhart, Shawn R.Abreo, EduardoLone, Rafiq A.Abrunhosa, LuísLu, GuodongAbuley, IsaacLu, LaifengAdamčík, SlavomírLuangsa-ard, Janet JenniferAddi, MohamedLubyanova, AlsuAdebayo, Elijah A.Lücking, RobertAdenis, AntoineLuczaj, LukaszAdil, MohdLuis Alberto, Madrigal-PerezAfanasenko, Olga SilvestrovnaLuo, Chao-XiAgriopoulou, SofiaLuo, HongliAguirre, JesúsLuo, YingfengAhmad, JavedLurie, SusanAhmad, SibtainLv, YangyongAhmed, NabeelLynch, Joseph P.Ahmed, Nishat HussainMa, LiangAhsan, TaswarMa, MingAit-El-Mokhtar, MohamedMa, RuonanAkbar, MuhammadMachado, MarinaAkcay, CaglarMachuca, ÁngelaAkhter, AdnanMacioszek, ViolettaAlam, Muhammad WaqarMadrid, MarisaAlaniz, SandraMagain, NicolasAlex, AnoopMagistà, DonatoAlexandrova, AlinaMagnano Di San Lio, GaetanoAlfandari, SergeMaguire, IvanaAl-Fatimi, MohamedMaharachchikumbura, SajeewaAl-Hatmi, Abdullah M. S.Mahboubi, AmirAli, ImranMahendra, ShailyAli, SajidMahmoudi, HassanAli, ShahidMahmoudi, ShahramAlisaac, EliasMaia, ThiagoAlizadeh, MarziehMajeed, ZahidAlizargar, JavadMalar, Mathu C.Almaguer, MichelMalfa, GiuseppeAlmeida, Marcos AbreuMałgorzata, ZiarnoAlmeida, Ricardo Sérgio CoutoMalik, AdeelAlonso-Monge, RebecaMalosso, ElaineAl-Qurashi, Adel D.Manamgoda, Dimuthu S.Alvarez Duarte, EduardoManawasinghe, Ishara S.Alves Junior, Sérgio LuizMang, Stefania MirelaAlves, ArturManjunathagowda, Dalasanuru ChandregowdaAlves, Clayton QueirozMannaa, MohamedAlves, RosanaMantadakis, ElpisAmara, AmroMantzoukas, SpiridonAmarowicz, RyszardManyes, LaraAmeen, FuadMaphanga, Tsidiso G.Amillis, SotirisMarangi, Giovanni FrancescoAn, LinnaMarcet-Hoiben, MarinaAnand, GautamMarchese, PietroAnastasopoulou, AmaliaMarcolongo, LoredanaAnco, Daniel J.Mareković, IvanaAnderson, AnneMaresi, GiorgioAndes, DavidMarino, AndreaAndrade, GaldinoMarius, NiculauaAndrade-Silva, LeonardoMarjanović, ŽaklinaAngelini, PaolaMarkina, Nadezhda M.Angulo, DavidMarkovskaja, SvetlanaAnjam, Muhammad ShahzadMarsaux, BenoîtAnkrah, Alfred O.Martin, RonnyAnnas, SallehMartínez Álvarez, José AscenciónAranda, AgustínMartínez Kopp, SebastiánAraújo, DanielaMartínez-Duncker, IvánArgyropoulos, Dimitris S.Martinez-Espinoza, AlfredoArikan-Akdagli, SevtapMartínez-Moreno, FernandoArroyo, JavierMartini, FlorenciaArsenijević, Valentina ArsićMartin-Vicente, AdelaArsenijevic, Valentina ArsicMascarin, Gabriel MouraArseniu, Anca-MariaMastore, MaristellaArunadevi, NatarajanMasuya, HayatoArya, Gulab ChandMatei, FlorentinaAsadzadeh, MohammadMatei, IoanaAshkenazi-Hoffnung, LiatMatny, OadiAsperges, ErikaMatočec, NevenAssyov, BorisMauve, CarolineAthanasiou, Labrini V.Mayo-Prieto, SaraAufrecht, JaydeMayser, PeterAvalos, JavierMazumdar, PurabiAvkan-Oğuz, VildanMazur, StanisławAydi-Ben-Abdallah, RaniaMazzaglia, AngeloAzinheira, Helena G.Mazzarello, VittorioBag, Manas KumarMcClelland, ErinBagagli, EduardoMcCormack, Joseph G.Bahr, Nathan C.McCormick, Susan P.Bahuguna, AshutoshMcCulley, Rebecca L.Bai, Feng-YanMcCulloch, Graham A.Baig, AtifMcMurray, MichaelBailão, Alexandre MeloMedel Ortiz, RosarioBakare, OladapoMedina, NardaBakhshi, MounesMedina, RocioBakonyi, JozsefMeijer, Eelco F. J.Balaeş, TiberiusMeijer, HaroldBalasubramani, Subramani ParanthamanMeis, JacquesBaldin, ClaraMelhem, MárciaBaldisserotto, AnnaMello, Thaís P.Balletto, ElisaMendes, Kassio F.Balois-Morales, RosendoMendes-Bastos, PedroBamisile, Bamisope SteveMerwid-Ląd, AnnaBanach, ArturMešić, ArminBandala, VictorMesterhazy, AkosBandara, SubhaniMestre Furlani, María VictoriaBaptista Rosas, Raúl CuauhtémocMeza-Carmen, VíctorBaraket, GhadaMichán, CarmenBaranova, EkaterinaMierzejewska, JolantaBaranowska, MarlenaMihál, IvanBarantsevich, Elena P.Mikkel Rank, NielsenBarbosa, RenanMilewski, SławomirBarchiesi, Francesco J.Millán-Chiu, Blanca EdithBar-Haim, ErezMiller, Andrew N.Baroncelli, RiccardoMillner, Patricia D.Barreno, EvaMillot, MarionBar-Yosef, Dana LaorMima, Ewerton GarciaBaschien, ChristianeMinerdi, DanielaBassi, DanieleMira, Nuno PereiraBassolé, Imaël Henri NestorMirabile, GiuliaBauer, IngoMisas, ElizabethBazaz, RohitMishra, AvinashBehiry, Said I.Missio, André LuizBehnke-Borowczyk, JolantaMitsuyama, SusumuBelanger, Faith C.Mittelmeier, WolframBelbahri, LassaâdMiura, ChihiroBellanger, Anne-PaulineMoazeni, MaryamBellet, VirginieMohali, Sari R.Bellmann, RomualdMohamed, El-SaadonyBen-Ami, RonenMohamed, GamalBenítez, ÁngelMohamed, HassanBenito, Ernesto P.Mohamed, Mahmoud S. M.Ben-Laouane, RajaMohammad, AqilahBentvelsen, RobbertMohammadi, HamidBenz, PhilippMolero, GloriaBerestetskiy, Alexander O.Monclaro, Antonielle VieiraBerger, Ralf G.Mondal, AlokBeric, TanjaMondello, VincenzoBerillo, DmitriyMonedeiro, FernandaBernadette-Emoke, TelekyMonod, MichelBernstein, NiritMontaño, Noé ManuelBerruyer, RomainMonteiro-Neto, ValérioBertolini, Maria CeliaMonteiro-Vitorello, Claudia BarrosBeyhan, SinemMora, Delio JoséBezerra, JadsonMoraes Gimenes Bosco, SandraBezirtzoglou, EugeniaMorales, DiegoBharudin, IzwanMora-Montes, Héctor M.Bhat, Aashaq HussainMorán-Diez, María E.Bhatia, NealMoreau, Pierre-ArthurBhatt, KalpanaMoreira, Silvino IntraBhattacharya, SomanonMoreno, María VirginiaBhunjun, Chitrabhanu S.Morganti, GiuliaBi, LuningMorgunov, IgorBi, YangMorovati, HamidBian, ZhuyunMorozova, Olga VBianchi, ElisabettaMorreale, GiacomoBianco, FrancescoMorrissey, Catherine OrlaBiel, WiolettaMoskovskiy, MaksimBielinsky, Anja KatrinMousa, Magdi A. A.Biketova, AlonaMouyna, IsabelleBillmyre, BlakeMroczyńska, MartynaBimšteine, GunitaMrsa, VladimirBinda, ElisaMucalo, AnaBinder, UlrikeMuggeo, PaolaBirch, Joseph D.Mukhin, Victor AndreevichBirsa, Mihail LucianMukhtar, TariqBjerketorp, JoakimMulas, MaurizioBłaszczyk, LidiaMundra, SunilBlecher, RonenMunir, ShahzadBliss, JosephMuñoz, JulianBoggian, KatiaMuntyan, Maria S.Bohu, TsingMurali, MahadevamurthyBölcskei, HedvigMuthu, ValliappanBonhomme, JulieMutturi, SarmaBonini-Domingos, Claudia R.Mysore, KeshavaBono, HidemasaNafie, Mohamed S.Boonyuen, NattawutNaganuma, TakeshiBorman, Andrew M.Nagl, MarkusBorovička, JanNagoth, Joseph AmruthrajBorymski, SławomirNaher, LailaBorza, TudorNai, Yu-ShinBotella, LeticiaNajafzadeh, Mohammad JavadBottone, Maria GraziaNamasivayam, S. Karthick RajaBouchara, Jean-PhilippeNante, NicolaBourret, TylerNapoleão, Thiago HenriqueBoutaj, HananeNarmani, AbolfazlBrandão, JoãoNarra, Hema PrasadBratek, ZoltánNascimbene, JuriBrayer Pereira, Daniela IsabelNasuno, RyoBroda, MagdalenaNatvig, Donald O.Brondani, MichelNavathe, SudhirBrooks, SiraprapaNawal, Abd El-BakyBrown, JasonNawrot, UrszulaBrowne, KatrinaNeal, TimothyBrunialti, GiorgioNechepurenko, Ivan V.Bruno, Giovanni L.Nechwatal, JanBruno, VincentNeely, MichaelBuchta, VladimirNegm, WalaaBudzynska, SylwiaNegri, MelyssaBui, Duc CuongNeiman, Aaron M.Buil, JochemNeli, Vilhelmova-IlievaBulet, PhilippeNelson, Rex T.Bultman, TomNemer, NabilBurcul, FrankoNeri, AndreaBurgess, TreenaNesler, AndreaBurmester, AnkeNeumann, Aaron K.Burrola-Aguilar, CristinaNg, SiewbeeButenko, Konstantin O.Ngamskulrungroj, PopchaiButler, GeraldineNguyen, Thuong T. T.Bzducha-Wróbel, AnnaNguyen, TuanCabanás, Carmen Gómez-LamaNickerson, KennethCaboň, MiroslavNicolas, Francisco E.Cabral, Tiara SousaNicoletti, RosarioCacciola, NunzioNiculae, MihaelaCacciola, Santa OlgaNieróbca, AnnaCáceres, Diego H.Nieva, Amira SusanaCaeiro, Maria FilomenaNigam, DeeptiCafaro, ValeriaNikolić, ValentinaCai, GuohongNikoloudakis, NikolaosCai, LeiNiño-Sánchez, JonathanCai, MenghaoNiño-Vega, Gustavo A.Cal, MagdalenaNkadimeng, SanahCalderón, Enrique J.Noman, AliCalderone, RichardNoman, EfaqCamacho, Irene Gomes CâmaraNoni, MariaCamargo, Mariana GuedesNoumeur, Sara R.Camargo-Ricalde, Sara LucíaNousheen, IqbalCamenzind, TessaNovak Frazer, LilyCan, HüseyinNowacka-Jechalke, NataliaCañas, José A.Nunes, Luiz R.Canito, Alessandra DiNúñez, Jose Alfonso DomínguezCánovas, DavidNunzi, EmiliaCantamessa, SimoneNuskern, LucijaCanteros, CristinaNwaiwu, OgueriCapece, AngelaOberheitmann, BorisCapó, VirginiaOcimati, WalterCardoso, PauloOda, KenCardoza, Rosa ElenaOgihara, JunCarrasco, JaimeOgórek, RafałCarter, Dee A.Ogundeji, Adepemi O.Cartmill, Andrew D.Oh, Seung-YoonCasalini, GiacomoOh, Soon-OkCasanova-Katny, AngélicaOhm, Robin A.Caspari, ThomasOhtomo, RyoCastagnola, ElioOkay, SezerCastell-Miller, ClaudiaOladele, Rita O.Castillo, LuisOlaru, Octavian TudorelCastrillo, DavidOliva, JonàsCastro, Jean FrancoOliveira, ManuelaCastro, Ricardo DiasOliveira, RuiCatana, RodicaOrasch, ThomasCazapal-Monteiro, Cristiana FilipaOrlikowska, TeresaCeci, AndreaOsherov, NirCelakovska, JarmilaOsiewacz, Heinz D.Černáková, LuciaOszako, TomaszCesaro, SimoneOtašević, SuzanaChacón-Hernández, Julio C.Oyardı, ÖzlemChakraborty, TanmoyÖz, YaseminChallagundla, LavanyaÖzdemir, Rahmi CanChandrasekar, PranatharthiÖzenci, VolkanChandrasekaran, MurugesanOzimek, EwaChang, Cheng-Wei TomÖzkan, SelinChao, Chien-MingPaciolla, CostantinoChappell, Thomas M.Pacioni, GiovanniCharpentier, ElénaPackeu, AnnChassot, FrancieliPaczos-Grzeda, EdytaChastain, DanielPadilla-Guerrero, Israel EnriqueChavez, FranciscoPadkina, Marina V.Chayakulkeeree, MetheePadoin, ChristopheCheedarala, Ravi KumarPadrão, JorgeChekanov, KonstantinPaetz, ChristianChen, ChangbinPaim, KennioChen, Chien-LiPais-Vieira, MiguelChen, Ko-HsuanPaiva, Diana SofiaChen, QianPalenzuela, José AntonioChen, ShaohuaPalermo, Anna MariaChen, TaixiangPallotto, CarloChen, WanhaoPallua, Johannes DominikusCheng, Ya-YunPalma, CarlaCheng, Yuan-BinPalmer, MichaelChiang, Yu-ChungPalmerin-Carreño, Dulce MariaChiba, SotaroPalmero-Llamas, DanielChieco, CamillaPandey, PiyushChoi, Jong SooPane, CatelloChoupina, Altino BrancoPanepinto, JohnChristova, Petya KoevaPańka, DariuszChung, Pei-ChePapakonstantinou, EvgeniaChutrakul, ChanikulPapp, ViktorCiarcia, RobertoParada, RodolfoĆilerdžić, JasminaParaskevi, MaraCilia, GiovanniPardo, Juan PabloĆirić, JelenaPardo-Giménez, ArturoCiudad, ToniPariona, NicolazaCláudia, GodinhoPark, Hee-SooCleary, IanParra, ClaudiaCockerell, Clay J.Pasechnik, TatyanaCoelho, Maria Alice ZarurPasmans, FrankCohen, Yigal R.Pasqualetti, MarcellaColom Valiente, María FranciscaPasquali, MatiasColombo, ArnaldoPassera, AlessandroConceição-Silva, FátimaPatel, GopalContesini, Fabiano JaresPatel, Jai SinghConti, HeatherPatel, RavikumarConti, StefaniaPatel, SanjayCooke, DavidPatkowska, ElżbietaCooper, Elizabeth A.Paukov, AlexanderCopolovici, Dana MariaPaul, Narayan ChandraCorcobado, TamaraPavlov, Igor N.Cordero, RadamesPawłowska, JuliaCordoba, Susana B.Pedrini, NicolásCorry, David B.Pedroso, Reginaldo Dos SantosCortés, Andrés J.Pekmezovic, MarinaCorti, CristinaPeng, MaoCosio, TerenzioPentimone, IsabellaCosoveanu, AndreeaPeralta-Ruiz, YeimmyCosta, DanielaPereira Borba-Santos, LuanaCosta-de-Oliveira, SofiaPereira, Antônio BatistaCovo, ShayPerera, Rekhani HansikaCoy-Barrera, EricssonPeres, Nalu T. A.Cozzi-Lepri, AlessandroPérez García, Luis AntonioCreamer, RebeccaPérez Martínez, SimónCrucitti, DalilaPerlin, Michael H.Crutz-Le Coq, Anne MariePernek, MilanCruz, CristinaPerrino, Enrico VitoCruz-Sosa, FranciscoPerrone, GiancarloCsernetics, ÁrpádPersat, FlorenceCuenca-Estrella, ManuelPertile, GiorgiaCuestas, María LujánPerveen, ShaguftaCui, HongzhuPeterson, MichaelCunha Zied, DiegoPfaller, MichaelCushion, Melanie T.Pfliegler, WalterCzislowski, ElizabethPhilipp, ChristianD’Ovidio, Maria ConcettaPiecuch, AgataDa Silva, Josué J.Piegza, MichałDa Silva, Luís C. N.Piesik, DariuszDa Silva, Thiago AparecidoPietrzak, WitoldDababat, AmerPila, Emmanuel A.Dal Forno, ManuelaPilarska, Agnieszka A.Dale, Jenna C. M.Piłsyk, SebastianDama, MuraliPimentel, Maria Ines F.Dannaoui, EricPineda-Hidalgo, Karen V.Das, AntuPino-Bodas, RaquelDas, KanadPinotti, LucianoDas, SubhaPinto, AnaDas, TaraprasadPircalabioru, GratielaDatta, KausikPlacido, JerssonDavolos, DomenicoPlasencia, JavierDavydenko, KaterynaPlaza Díaz, JulioDayarathne, MonikaPleckaityte, MildaDe Aguiar Cordeiro, RossanaPletsa, VassilikiDe Alteriis, ElisabettaPobiega, KatarzynaDe Andrade, Adalgisa RodriguesPoddighe, DimitriDe Andrade, Cristiano J.Poitevin, Carolina G.De Araujo, Jackson VictorPolacheck, ItzhackDe Azevedo Melo, Analy SallesPolčic, PeterDe Cock, HansPoli, AnnaDe Curtis, FilippoPollastro, StefaniaDe Farias, RobsonPombeiro-Sponchiado, Sandra Regina C.De Francia, SilviaPomraning, KyleDe J. Treviño-Rangel, RogelioPopescu, Sorina C.De La Cruz, David RodríguezPopovici, VioletaDe La Rosa-García, SusanaPortilla, MaribelDe La Varga, HerminiaPortillo-Estrada, MiguelDe Matteis, ValeriaPozdnyakova, NataliaDe Miccolis Angelini, Rita MilviaPrabhakar, AditiDe Moraes, Daniel ClementePrakash, HariprasathDe Musis, CristianaPramanik, DibyajyotiDe Oliveira, AlbertoPrasanna, RadhaDe Souza Baptista, Andrea ReginaPrasannakumar, N. R.De Souza Ramos, LíviaPrathumpai, WaiDe Victoria, Filipe CarvalhoPrigione, ValeriaDeakin, GregPripdeevech, PatchareeDecock, ConyPrisa, DomenicoDeepe, GeorgePrista, CatarinaDegola, FrancescaPromdonkoy, BoonhiangDel Mar Hernández, MaríaPrzybyłko, Sebastiandel Olmo, Marcel LíPudake, RameshDel Pilar Jimenez, MariaPutzke, JairDel Pilar Ortega-Larrocea, MaríaPůža, VladimírDela Cruz, Thomas EdisonQader, MalliqueDemanche, ChristineQin, JiaoDeng, ShuwenQueiroz-Telles, FlavioDeresinski, Stanley C.Quiroga, DiegoDerntl, ChristianRåberg, LarsDesgarennes, DamarisRacovita, RaduDeshpande, Mukund V.Radu, Nicoleta NicoleDesoubeaux, GuillaumeRahnama, KamranDi Filippo, MarziaRai, RikkyDi Gennaro, FrancescoRaimondo, Maria LuisaDi Martino, CatelloRaja, RathinamDiaz-Minguez, José MaríaRajchenberg, MarioDilfuza, EgamberdievaRamalingam, SrinivasanDinos, George P.Ramesh Sundar, AmalrajDiongue, KhadimRamírez Soto, Max CarlosDitta, AllahRamirez-Castrillon, MauricioDivashuk, MikhailRamirez-Tapias, Yuly A.Dlauchy, DenesRapala-Kozik, MariaDługaszewska, JolantaRasiukevičiūtė, NeringaDobias, RadimRaudaskoski, MarjattaDoilom, MingkwanRauseo, Adriana M.Dojnov, BiljanaRaymundo, TaniaDombradi, ViktorRazaq, AbdulDomizio, PaolaRecalcati, SebastianoDong, CaihongReeves, EmerDonnelley, Monica A.Relic, DubravkaDonovan, FaribaRementeria, AitorDos Santos, André Luis SouzaReuter, KlausDouira, AllalReveglia, PierluigiDoyle, SeanRexer, Karl-HeinzDu Plessis, Heinrich WReyes Montes, RocioDuarte, RicardoReyes, FernandoDuarte-Escalante, EsperanzaRezaei-Matehkolaei, AliDufossé, LaurentRia, FrancescoDufresne, PhilippeRibeiro, Martha SimõesDumitru, Irina MagdalenaRiccioni, ClaudiaDurán-Peña, María JesúsRice, Steven J.Dylag, MariuszRichardson, RiinaDzikowska, AgnieszkaRickerts, VolkerEbel, FrankRiemann, MichaelEbinezer, Leonard BarnabasRigling, DanielEconomou, Christina N.Rincón-Enríquez, GabrielEdit, FarkasRinker, Danny LeeEfremenkova, Olga VRinnerthaler, MarkEgerton-Warburton, LouiseRipa, MatteoEkprasert, JindaratRisco-Castillo, VeronicaEl Khalloufi, FatimaRisdian, ChandraEL Moussaoui, AbdelfattahRivas Franco, FedericoElhady, SamehRivas, FatimaEliopoulos, Panagiotis A.Rizvi, Syed A. A.El-Seedi, HeshamRo, Hyeon-SuElshafie, Hazem S.Robak, MałgorzataElsharkawy, MohsenRoberts, Elizabeth LewisElsherbiny, ElsherbinyRoche, BenjaminEl-Shetehy, MohamedRodemann, BerndEl-Tarabily, KhaledRodrigues Marques, João PauloElwakil, BassmaRodrigues, Célia F.Enz, Andreas EugenRodrigues, JoanaErcisli, SezaiRodriguez- Cerdeira, CarmenErdoğan, AyşegülRodriguez Vazquez De Aldana, BeatrizEscobedo-Bonilla, Cesar MarcialRodríguez-Romero, JulioEsher, ShannonRodríguez-Temporal, DavidEspeso, Eduardo A.Rogers, ThomasEspinel-Ingroff, Ana V.Rolle, LucaEspinosa-Ortiz, ErikaRollin-Pinheiro, RodrigoEsqueda-Valle, MartinRolo, JoanaEsquivel-Naranjo, EdgardoRomagnoli, RomeoEsteves, Ana CristinaRomano, PatriziaEtxebeste, OierRoncero, CesarEvangelista De Oliveira, Manoel MarquesRopelewska, EwaFachin, Ana LúciaRosas-Taraco, AdrianFaedda, RobertoRosenau, FrankFaizulin, AlexanderRosendahl, SørenFakhim, HamedRossi, Franco RubénFan, JingRoy, SwarnenduFarcasanu, Ileana C.Rubini, AndreaFarina, ClaudioRubio, BelénFarkas, Edit É.Rudnóy, SzabolcsFarooq, TahirRuiu, LucaFarooqi, JoveriaRuiz-Baca, EstelaFasciana, TeresaRusinek, RobertFast, Naomi M.Saade, AnastasiaFattahi, AzamSabater-Muñoz, BeatrizFaulds, CraigSabel, TinaFelczak, AleksandraSaberi-Riseh, RoohallahFeng, MingguangSabino, RaquelFernandes, ÉvertonSabou, MarcelaFernandes, LarissaSabry, Bassem A.Fernández, Francisco J.Sácký, JanFernandez, JessieSá-Correia, IsabelFernandez-Garcia, RaquelSadamoto, SotaFerrante, PatriziaSadykova, VeraFerreira, MarceloSaito, MasanoriFerreira-Paim, KennioSaito, SeiyaFerreiro-González, MartaSalamov, AsafFierer, JoshuaSalbitani, GiovannaFioriti, SimonaSallam, Ahmed B.Fischer, JochenSalmanton-García, JonFiuza, Patrícia OliveiraSalvi, PrafullFlorek, MagdalenaSamaras, AnastasiosFlores, CarolinaSampaio, PaulaFloudas, DimitriosSamsonova, MariaFogarasi, MelindaSanchez Fernandez, Rosa ElviraFolch-Mallol, Jorge L.Sánchez Torres, PalomaFontaine, ThierrySanchez, YolandaFontaine, VéroniqueSánchez-Cruz, RicardoFonte, LuisSánchez-Hernández, EvaForster, JohannesSánchez-Quitian, Zilpa-AdrianaFortún, JesúsSangabriel-Conde, WendyFouda, AmrSano, AyakoFoulongne-Oriol, MarieSantacroce, LuigiFournier, GregorySantiago-Tirado, Felipe H.Fox, AaronSantos, LilianaFradin, ChantalSar, TanerFrancis, FredericSarwar, SaminaFrancisco, Elaine CristinaSatoh, KazuoFranco-Duarte, RicardoSavoie, JeanFrases, SusanaSayed, Samy Mahmoud H.Free, StephenSbaraini, NicolauFregeneda-Grandes, Juan-MiguelSchauwvlieghe, AlexanderFreitag, JohannesSchipper, KerstinFrías-De-León, María GuadalupeSchirtzinger, Erin E.Fuchs, BethSchmidt, MartinFuentes-Davila, GuillermoSchmidt, OlafFukuda, MakihaSchoonbeek, Henk-janGabriel, IwonaSchouten, AlexanderGago, SaraSchroeckh, VolkerGai, YunpengSchüller, Hans-JoachimGalgiani, John N.Schwarz, PatrickGamaletsou, Maria N.Schweigkofler, WolfgangGanesan, ArasuSecenji, AleksandarGanesan, KumarSekrecka-Belniak, AnnaGangneux, Jean-PierreSemenov, Mikhail V.Gannibal, Philipp B.Semper, CameronGao, JiangyunSénéchal, FabienGarau, GiovanniSenik, SvetlanaGarcia-Clemente, MartaSenn-Irlet, BeatriceGarcia-Descalzo, LauraSenthilkumar, MurugaiyanGarcia-Effron, GuillermoSeok, HyeriGarcía-Guzmán, MaríaSerce, Cigdem UlubasGaribay-Orijel, RobertoSergeeva, YanaGarre, VictorianoSergio, Pérez-OrtegaGarrido, Paula MelisaServín Garcidueñas, Luis EduardoGarrido-Jurado, InmaculadaSetianingrum, FindraGarrigues, SandraSevcikova, HanaGarvy, Beth A.Sexton, Joseph D.Gautam, VibhavShah, SujitGbashi, SefaterShaik, Haq AbdulGe, ZaiweiShakeri, AbolfazlGea-Banacloche, Juan CarlosShalabi, AhmedGebremariam, TeclegiorgisShang, ZhuoGelli, AngieSharma, AnupamGené, JosepaSharma, MinaxiGeorgieva, MargaritaSharma, Tilak RajGerin, DonatoShastry, Rajesh P.Ghazanfar, Muhammad UsmanShcherbakova, ViktoriaGhobad-Nejhad, MasoomehShemer, AvnerGhosta, YoubertSheteiwy, MohamedGiangrieco, IvanaShetty, Kateel G.Giosa, DomenicoShida, YosukeGirich, Elena V.Shin, Kwang-SooGits-Muselli, MaudShiryaev, Anton G.Giurgiulescu, LiviuShoukat, Rana FartabGiusiano, GustavoShrestha, PrachandGkizi, DanaiShu, CanweiGlamočlija, JasminaShubitz, Lisa F.Glen, MoragSiede, WolframGnat, SebastianSiless, GastónGobin, IvanaŠilha, DavidGodoy, LilianaSilva, Fábio Sérgio Barbosa DaGohari, GholamrezaSilvestrini, LuciaGöhre, VeraSimeonov, VasilGolo, PatriciaSimoneau, PhilippeGolubeva, TatianaSimonetti, GiovannaGomes, AnitaSimonic-Kocijan, SuncanaGomes, FernandaSimonini, GiampaoloGomes, SóniaSingh, AbhijeetGómez, Carlos HernandoSingh, AmbrishGomi, KatsuyaSingh, GopalGonçalves, MicaelSingh, ShaktiGonçalves, Pedro AntonioSingh, SukhvinderGonçalves, Sarah SantosSingla, NidhiGonçalves, TeresaSionov, RonitGonda, SándorSipsas, Nikolaos V.Gong, ZhiyongŠirić, IvanGonzález Estrada, Ramsés RamónSjölin, JanGonzález García, VicenteSłaba, MirosławaGonzalez, AngelSnelders, EvelineGonzález-Teuber, MarciaSoeprobowati, Tri RetnaningsihGopal, JudySohrabi, MohammadGóral, TomaszSolano, CarlosGoršek, AndrejaSolano, FranciscoGospodarek, JaninaSolís García Del Pozo, JuliánGrabarczyk, MałgorzataSomerville, TobiGraciano De Paula, RenatoSomma, StefaniaGrande Tovar, Carlos DavidSong, XiaoshuangGranica, SebastianSophianopoulou, VickyGreb-Markiewicz, BeataSorokan, AntoninaGreen, SarahSorrentino, AngelaGremiao, Isabella Dib FerreiraSosa, María CristinaGresnigt, MarkSoto-Suárez, MauricioGriffith, Gareth W.Sousa Dos Santos, José CleitonGrizanova, Ekaterina V.Soytong, KasemGroll, Andreas H.Spina, FedericaGruhn, GeraldSpinelli, VeronicaGrupe, ArthurSpisni, AlbertoGrzywacz, BeataSridhar, Kandikere R.Gu, ChenjianSrikanth, KrishnamoorthyGuan, XinSrisuttiyakorn, ChutikaGuegan, HélèneStaats, Charley ChristianGueidan, CécileStan, LauraGuera, AlfredoStasińska, MałgorzataGuerra-Sanchez, GuadalupeStefanini, IreneGuerriero, IlariaStefaniuk, DawidGülmez, DolunaySteinbrink, Julie M.Guo, JinhuSteindorff, AndreiGusella, GiorgioSteinmann, JoergGusev, EvgeniiStępień, ŁukaszGusmão, Luis Fernando PascholatiStevens, PhilipGutierrez Martinez, PorfirioStoian, VladGuzmán Dávalos, LauraStrakhovskaya, MarinaHadebe, SabeloStuart, David T.Haelewaters, DannyStudt, LenaHage, Chadi A.Sturtevant, JoyHamada, YukihiroSu, Chih-ChiaHamberg, LeenaSu, YingjieHameed, KhalidSubhan, MishalHan, BingSubramaniam, RajagopalHan, QinqinSubramanian, SowmyalakshmiHaneke, EckartSuchodolski, JakubHansen, KarenSuleiman, Waleed BakryHantula, JarkkoSulej, JustynaHarding, MichaelSułek, AlicjaHarel, Yael MellerSummers, Katie LynnHarrison, Paul H. M.Sun, FengjieHartmann, AntonSun, Jing-ZuHashem, MohamedSun, Pei-LunHassan, ErrolSun, XiangHassan, Saad El-DinSunpapao, AnuragHay, RoderickSuprun, Andrey R.He, BinSutton, GrangerHe, FeiSuwannarach, NakarinHe, MingyuSuzuki, NobuhiroHeczko, Piotr B.Svantesson, StenHedayati, Mohammad T.Svetec, Ivan KrešimirHeidari, ArashSvidzinski, Terezinha Inez EstivaletHeinisch, Jürgen J.Sweany, RebeccaHenrique Costa, JonasSwenie, Rachel A.Henry, Ajuna B.Swietnicki, WieslawHermosa, RosaSyed, KhajamohiddinHernández-Domínguez, Eric EdmundoSylla, KhadimeHernández-Ochoa, LeonSyromyatnikov, Mikhail Y.Herrera, Erick MartínezSysouphanthong, PhongeunHerrera, HectorSzczech, MagdalenaHerrera, MartaSzebenyi, CsillaHerrera-Estrella, AlfredoSzepietowski, JacekHewedy, OmarSzigeti, Zsuzsa MáthénéHicham, ElmsellemSzpyrka, EwaHidalgo Bucheli, WilliamSzultka-Młyńska, MałgorzataHiguchi, YujiroSzweda, PiotrHilário, SandraTaborda, Carlos PelleschiHillström, LarsTadahiro, SuzukiHines, Justin K.Talento, AlidaHise, Amy G.Talhinhas, PedroHoffmann, Klaus H.Tan, Ban HockHönigl, MartinTan, HaoHorin, Tal BenTan, Yee ShinHorwitz, Benjamin A.Tanaka, MizukiHorwitz, BenjamineTangerina, Marcelo Marucci PereiraHosen, IqbalTanguay, PhilippeHoshino, TamotsuTapia, EduardoHossain, EkramTarasco, EustachioHoving, Jennifer ClaireTarca, ElenaHoyer, Katrina K.Tarighi, SaeedHu, Dian-MingTariq, MohsinHu, HaifeiTarman, KustiariyahHu, JieTashima, ToshihikoHu, QiongboTavares, Aldo HenriqueHu, XueboTay, Sun TeeHu, YoucaiTeixeira, Miguel CachoHuang, JunTéllez-Téllez, MauraHubka, VitTenor, Jennifer L.Humar, MihaTeparić, RenataHurley, Jennifer M.Terzi, ValeriaHurraß, JuliaThambugala, KasunHusain, MureedThannhauser, Theodore W.Husen, AzamalThaochan, NaritHussain, AbidThevi Selvarajah, GayathriHussain, MubasherThomaz, DaniloHussain, ShahThongbai, BenjarongHyskova, VeronikaTibpromma, SaowaluckIbrahim, SabrinTimmons, LisaIgnatov, AlexanderTirado-Sánchez, AndrésImbert, SébastienTisi, RenataIngram-Smith, CherylTkaczyk, MiłoszIntra, JariTokarev, YuriIoannou, PetrosToledo, AndreaIriart, XavierTomko, Robert J.Isfahani, Mehdi NasrTomoda, HiroshiIslam, MazharulTongdeesoontorn, WirongrongIslam, Md RabiulTopalidou, EleniIslam, Md. AmirulToriello, ConchitaIsmaiel, Adnan A.Torres, RodrigoIturriaga, TeresaTorres-Mendoza, DanielIturrieta-González, Isabel AntonietaTóth, BrigittaIwaniuk, PiotrTóth, GergőJabeen, KausarTrabasso, PlinioJabes, Daniela L.Tran, Phu-TriJacob, ShreyTran, Van-TuanJadon, Kuldeep SinghTrdan, StanislavJain, Bhawik KumarTrifan, AdrianaJain, IraTripathi, JitendraJakšić, DanielaTrombitas, Veronica ElenaJalal, AbdulTrong Tuan, NguyenJames, PeterTrovão, JoãoJankowiak, RobertTrzewik, AleksandraJantasuriyarat, ChatchawanTsai, Ming-HorngJanusz, GrzegorzTsang, Chi-ChingJarerat, AmnatTsivileva, OlgaJaronski, Stefan T.Tsui, Clement K. M.Jaroszuk-Ściseł, JolantaTupaki-Sreepurna, AnanyaJavidnia, JavadTvrdá, EvaJayasiri, Subashini ChathuminiTziros, George T.Jayawardena, Ruvishika ShehaliUdayashankar, Arakere ChunchegowdaJean-Luc, JanyUehling, JessieJegadeesh, RamanUl-Hassan, ZahoorJevtić, RadivojeUllberg, MånsJha, Chaitanya KumarUlrich, SebastianJiang, YingUmar, MuhammadJiang, ZideUmeyama, TakashiJohansson, BjörnUmezawa, KiwamuJohnson, John A.Ungureanu, NicoletaJohnson, LindaUnk, IldikóJones, Gareth E. B.Urbez-Torres, Jose RamonJoniec, JolantaUrbina, HectorJorda, AnselmVadkertiova, RenataJouda, Jean-BoscoVainikka, AnssiJu, JianhuaVaksmaa, AnnikaJuan Garcia, CristinaValenčáková, AlexandraJumpponen, AriValenzuela-Lopez, NicomedesKaczmarek, AgataValera, María JoséKafarski, PawelValero, ClaraKakabouki, IoannaVali, GaborKakumyan, PattanaValiante, VitoKalkum, MarkusValiuškaitė, AlmaKamal, ShwetVan Den Nest, MiriamKaminska, JoannaVan Der Heyden, HervéKamou, NathalieVan Der Nest, Magriet A.Kane, Patricia M.Van Rhijn, NormanKanematsu, HideyukiVargas, SergioKano, RuiVasaitis, RimvysKara, Mehtap P.Vasić, Tanja P.Karabegovic, Ivana TVasic, VericaKarakaya, AzizVazquez, Jose A.Karaman, MajaVazquez-Hernandez, MariaKaraś, MagdalenaVázquez-Marrufo, GerardoKarbancioglu-Guler, FundaVázquez-Ovando, AlfredoKarkowska-Kuleta, JustynaVear, FelicityKarpov, DmitryVeerana, MayuraKarunarathna, Samantha C.Vek, ViljemKashyap, Prem LalVelikova, TsvetelinaKasianov, Artem S.Vellinga, ElseKatou, ShadiVeloso, JavierKatzilakis, NikolaosVenanzi, RacheleKaufman, David A.Venditti, MarioKaur, Jan NaseerVenieraki, AnastasiaKaushik, PrashantVeraldi, StefanoKaygusuz, Oguzhan KaygusuzVergara, Pablo M.Kazartsev, Igor A.Vergidis, PaschalisKebede, AidaVergine, MarziaKebert, MarkoVerma, PradeepKeighley, CaitlinVernelen, KrisKeller-Przybyłkowicz, SylwiaVianna, María FlorenciaKelly, StevenViapiana, AgnieszkaKenerley, CharlesVicari, MarkKeyhani, Nemat O.Vidican, RoxanaKhadka, RamVijayvargiya, PrakharKhalaf, Roy A.Villarino, MariaKhalifa, Mahmoud E.Villa-Tanaca, LourdesKhalil Bagy, Hadeel M. M.Villena, GrettyKhalil, Ahmed Mohamed AliViswanathan, RasappaKhan, Amer HayatVitale, RoxanaKhan, Faiz UllahVlainić, JosipaKhan, FazlurrahmanVlasák, JosefKhan, MasoodVon Wallbrunn, ChristianKhanh, Tran DangVondrák, JanKhayi, SlimaneVongsangnak, WanwipaKhilyas, Irina V.Voynikov, YulianKhunnamwong, PannidaVrioni, GeorgiaKhuong, Nguyen QuocWachowska, UrszulaKieliszek, MarekWakil, WaqasKijpornyongpan, TeeratasWakuliński, WojciechKim, Jung-EunWanasinghe, DhanushkaKim, KyoungtaeWang, GaofengKim, Min-JiWang, JianfengKim, Sung-KunWang, JieKin, KoryuWang, LipingKiran, MunazzaWang, MingyuanKirkland, Theo N.Wang, Myeong-HyeonKiss, AttilaWang, QimingKlassen, RolandWang, WenjieKlassert, Tilman E.Wang, ZhengKlaus, AnitaWarris, AdiliaKlepzig, Kier D.Watanabe, AkiraKnight, John MorganWatanabe, KyokoKnorre, Dmitry A.Wei, JunhongKobkul, LaotengWei, YongjunKobmoo, NoppolWengenack, NancyKoch, ChristianWhite, John KerrKocsis, TamásWidemann, EmilieKoide, Roger T.Widiastuti, AniKolomytseva, MarinaWidyowati, RetnoKominek, JacekWiederhold, NathanKonaté, KiessounWiewióra, BarbaraKononenko, Neonila V.Wijayawardene, NalinKontoyiannis, DimitriosWilgan, RobinKordalewska, MilenaWilk, MateuszKornitzer, DanielWilkinson, DerekKőrösi, LászlóWilliams, StephenKos, JovanaWilmes, DunjaKoschorreck, KatjaWilson, Andi M.Kosmidis, ChrisWińska, KatarzynaKosová, KláraWirth, FernandaKot, Anna MariaWisitrassameewong, KomsitKottom, Theodore J.Woods, Jon P.Koudounas, KonstantinosWoods, Marion L.Kouokam, Joseph CalvinWöstemeyer, JohannesKovacs, RenatoWozniak, KarenKovanda, Laura L.Wronska, Anna KatarzynaKowalski, TadeuszWu, Chi-JungKöycü, Nagehan DesenWu, PeideKozubowski, LukaszWu, Ting-ShuKrabel, DorisWurster, SebastianKraichak, EkaphanWyszkowska, JadwigaKrappmann, SvenXess, ImmaculataKraševec, NadaXia, ChaoKrasnopolskaya, LarissaXiaojie, WangKratosova, GabirlaXie, JiaqinKrier, FrançoisXie, XiananKrochmal-Marczak, BarbaraXie, ZexiongKról, Ewa DorotaXiong, JiaKruszewska, JoannaXu, JianpingKrysenko, SergiiXu, JinrongKryštofová, SvetlanaXu, MenglongKučera, ViktorXu, QingyanKulakovskaya, Tatiana V.Xue, ChaoyangKumar, AjayYamamoto, KoheiKumar, AundyYan, ShuoKumar, AwanishYang, KunlongKumar, JiteshYang, LeiKumar, K. J. SenthilYao, ShixiangKumar, ManuYaoita, YasunoriKumar, PradeepYehia, RamyKumar, PrasunYetmar, ZacharyKumar, RavinderYilmaz, AsliKumar, VinodYin, ChengmiaoKumaresan, PappanaickenYin, YanbinKumavath, RanjithYoda, TsuyoshiKušan, IvanaYoko Hirooka, ElisaKushnirov, Vitaly V.Young, Jo Anne H.Kwiatek, AgnieszkaYoussef, Sahar A.Labuda, RomanYu, Fu-QiangLachance, Marc-AndréYu, YangLafond, MickaëlYu, ZefenŁagowski, DominikYurchenko, EkaterinaLagrou, KatrienZabalgogeazcoa, ÍñigoLahlali, RachidZafar, AfiaLai, OlimpiaZaini, PauloLai, Wen-LinZamani-Noor, NazaninLake, Douglas F.Zambolim, LaércioLam-ubol, AroonwanZancopé-Oliveira, Rosely MariaLandgraf, DirkZanetti, StefaniaLang, ChristineZangeneh, Tirdad T.Langer, Ewald JohannesZapora, EwaLaraba, ImaneZara, SeverinoLaranjo, MartaZarrinfar, HosseinLatté, Klaus PeterZdybicka-Barabas, AgnieszkaLauberts, MarisZehra, AndleebLavergne, Rose-AnneZeng, HongmeiLe, Minh VienZeng, ZhaoqingLeclerque, AndreasZerva, AnastasiaLee, Ching-FuZhan, FangdongLee, ChowZhang, BangxiLee, HwayongZhang, HuanLee, Hyang BurmZhang, HuangLee, Marcia R.Zhang, JianhuaLee, Miin-HueyZhang, JiweiLee, Moo-SeungZhang, JiyeLee, Seng HuaZhang, KaiLee, Seung-hyoZhang, NiyaLee, Seung-YeolZhang, PanLee, Sun MiZhang, QingLegan, Andrew WesleyZhang, XianghuiLeitão, Jorge H.Zhang, XiaodongLemes, Ailton CesarZhang, YinglaoLešnik, SamoZhang, YongLevy, StevenZhao, ChanglinLi, GuotianZhao, ShuaiLi, HongminZhao, YuLi, LiZhao, ZhenzhenLi, XueZheng, LuLi, YanchunZheng, PingLimón, CarmenZhong, ZhenhuiLin, Hong-Ting VictorZhou, JunLin, Hung-YinZia, MohammadaliLin, Jun-FangZieliński, MarcinLin, Kuo-ChihZieniuk, BartłomiejLin, Shang YiZifcakova, LuciaLin, Wan-RouZiko, LailaLinaldeddu, Benedetto T.Živić, MiroslavLinkies, AdaZjalić, SlavenLipke, PeterZoll, JanLiporagi-Lopes, Livia CristinaZommiti, MohamedLipşa, Florin DanielZuccaro, GaetanoLisek, JerzyZucconi, LauraLiu, Biing HuiZuena, MartinaLiu, DongZulhendri, Felix


